# Gut microbiota–mitochondria–barrier–multiorgan axis: a network-based hypothesis for systemic injury

**DOI:** 10.3389/fimmu.2026.1885379

**Published:** 2026-08-20

**Authors:** Yachao Li, Huaijue Qiu, Xiang Gao, Yanling Zhang, Qin Xia

**Affiliations:** 1 Sichuan Academy of Medical Sciences, University of Electronic Science and Technology of China, and Sichuan Provincial People’s Hospital Operating Room, Chengdu, China; 2 School of Medicine, University of Electronic Science and Technology of China, Chengdu, China; 3 Department of Gastrointestinal Surgery, Sichuan Academy of Medical Sciences & Sichuan Provincial People’s Hospital, University of Electronic Science and Technology of China, Chengdu, China; 4 Department of Operating Room, Sichuan Academy of Medical Sciences & Sichuan Provincial People’s Hospital, University of Electronic Science and Technology of China, Chengdu, China

**Keywords:** gut microbiota, intestinal barrier, mitochondrial damps, mitochondrial dysfunction, multiple organ dysfunction syndrome, systemic inflammation

## Abstract

Intestinal barrier failure is considered a driver of multiple organ dysfunction syndrome (MODS) in critical illness; however, the precise molecular mechanisms linking altered gut microbes to systemic injury remain unclear. Here we propose a testable hypothesis: mitochondrial dysfunction within intestinal epithelial cells (IECs) acts as a mechanistic hub connecting microbial dysbiosis to barrier breakdown and eventual multiorgan damage. This system is not a unidirectional chain but a highly network-based process with bidirectional feedback loops, context-dependent interactions, and hypothetical cross-talk that require experimental validation. We review evidence that microbial metabolites—short-chain fatty acids, hydrogen sulfide, secondary bile acids—directly modulate mitochondrial respiration, membrane potential, and reactive oxygen species (ROS) production in the gut lining. Once mitochondrial quality control falters, the cell suffers adenosine triphosphate (ATP) depletion, excessive ROS, calcium-driven calpain activation, and leakage of mitochondrial damage-associated molecular patterns (mtDAMPs) like mitochondrial DNA (mtDNA) into the cytoplasm. These mtDAMPs ignite the cyclic GMP-AMP synthase (cGAS)-stimulator of interferon genes (STING) pathway and the NLR family pyrin domain containing 3 (NLRP3) inflammasome, disrupting tight junctions and converting the intestinal barrier into an active inflammatory broadcaster. Critically, the inflammatory response is amplified by immune intermediary layers, including neutrophil extracellular trap (NET) formation, monocyte/macrophage metabolic reprogramming, endothelial activation, and complement activation, which bridge local barrier disruption to systemic organ injury. When mtDAMPs reach the circulation, they trigger sterile inflammation in distant organs through shared innate immune sensors (Toll-like receptor 9 [TLR9] and NLRP3) in liver, lung, and brain. A word of caution: circulating mtDNA originates from multiple tissues (e.g., immune cells, skeletal muscle, liver) and therefore represents a systemic DAMP, not a gut-specific signal; its interpretation requires contextual information on tissue origin and temporal dynamics. Human evidence still lacks clear answers on temporal order, directionality, epigenetic mediation, and quantitative thresholds. We discuss how circulating metabolites and mtDNA could serve as candidate monitoring biomarkers to turn this conceptual network into a testable, quantitative model. Finally, we outline a multi-dimensional research framework (metabolite replenishment, mitophagy enhancement, epigenetic tuning) while stressing that any clinical application must await prospective validation. For now, the “microbiota–mitochondria–barrier–multiorgan” axis should be seen as a well-grounded hypothesis, not an established fact.

## 1 Introduction

Multiple organ dysfunction syndrome (MODS) remains a leading cause of death in intensive care units (1, 2). Despite better resuscitation and organ support, once MODS is fully established, few therapies can reverse it (3). That sobering reality has pushed researchers to look earlier in the injury cascade. Among the earliest suspects is the “leaky gut”—disruption of the intestinal barrier—which has been repeatedly linked to sepsis, systemic inflammation, and failure of distant organs (4, 5). Yet a lingering question remains unanswered: through exactly which intracellular events do gut microbes and their metabolites turn a local barrier defect into a whole-body inflammatory storm?

It is important to distinguish among four types of relationships in this system: (1) direct causal evidence (e.g., microbial metabolites affecting mitochondrial respiration), (2) reciprocal regulation (e.g., mitochondrial function feeding back to alter the luminal environment), (3) positive and negative feedback loops (e.g., inflammation impairing mitochondrial function, which in turn amplifies inflammation), and (4) hypothetical cross-talk that currently lacks direct experimental support (e.g., epigenetic mediation in human IECs). These distinctions are essential for interpreting the evidence discussed below.

Early work focused heavily on dysbiosis and elevated blood endotoxin levels, framing barrier failure as passive leakage of large molecules such as lipopolysaccharide (4). This model is simple, but it cannot explain why many patients without detectable bacterial translocation still develop systemic inflammation and multiorgan damage (3, 5). Over the past decade, an organelle seemingly far removed from the gut—the mitochondrion—has moved centre stage. Mitochondria in IECs do more than make ATP; they also sense microbial signals and can ignite inflammatory cascades (1, 2). Microbial metabolites such as short-chain fatty acids (SCFAs) and hydrogen sulfide (H_2_S) directly alter respiratory chain efficiency, membrane potential, and mitochondrial ROS (mtROS) production (1, 2). And when mitophagy—the cell’s quality-control system for mitochondria—fails, damaged mitochondria release danger signals like mtDNA, which can amplify local oxidative stress into systemic inflammation through innate immune pathways (1, 2).

**Figure 1:**
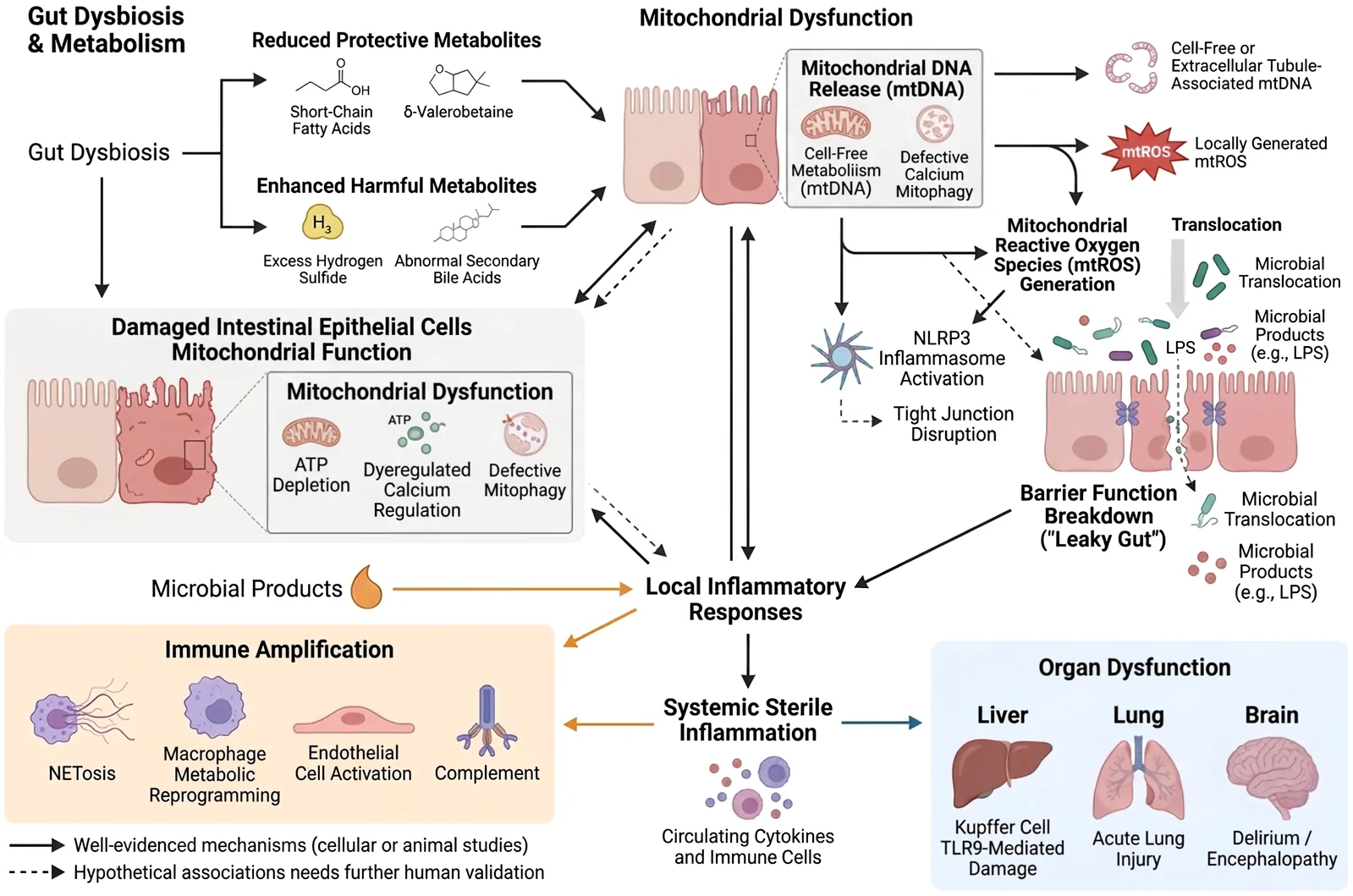
A network-based model of the microbiota–mitochondria–barrier–multiorgan axis in critical illness. Dysbiosis reduces protective metabolites (SCFAs, δ-valerobetaine) and promotes harmful ones (excess H_2_S, abnormal secondary bile acids). These metabolic shifts impair mitochondrial function in intestinal epithelial cells, causing ATP depletion, calcium dysregulation, and defective mitophagy. Damaged mitochondria release mtDNA into the cytoplasm and generate mtROS locally. mtDNA (a systemic DAMP) and locally generated mtROS activate the NLRP3 inflammasome and disrupt tight junctions, leading to barrier breakdown (“leaky gut”). Local mtDAMP release triggers inflammatory responses that are amplified by immune intermediary mechanisms (NETosis, macrophage metabolic reprogramming, endothelial activation, and complement), which propagate systemic sterile inflammation. Circulating mtDNA subsequently drives organ dysfunction—in the liver via Kupffer cell TLR9, in the lung as acute injury, and in the brain as delirium/encephalopathy. A positive feedback loop between mitochondrial damage and local inflammation amplifies the entire network. Solid arrows indicate mechanisms supported by substantial evidence (primarily from cell and animal studies); dashed arrows indicate hypothesized links that require further validation in humans. Orange arrows denote immune amplification; blue arrows indicate organ-specific effects. This framework emphasizes dynamic bidirectional interactions, distinct roles for mtDNA and mtROS, and immune amplification as an integrative bridge.

Putting these observations together, we argue for a new framework: mitochondrial dysfunction is not a bystander in barrier failure but a mechanistic hub linking upstream microbial imbalance to downstream remote organ damage. However, we emphasize that this is not a simple linear cascade; rather, it is a dynamic network with reciprocal interactions. For example, mitochondrial function can reciprocally regulate gut microbiota composition via changes in epithelial oxygen consumption and antimicrobial peptide secretion; inflammatory states can reshape metabolite profiles; and barrier disruption and immune activation form self-amplifying feedback loops. We present this model as a testable hypothesis, not an established mechanism. In this review we synthesise recent literature on gut microbiota–mitochondria crosstalk in barrier failure and systemic injury. We cover: how microbial metabolites regulate mitochondrial function in gut epithelial cells; how mitochondrial dysfunction disrupts tight junctions and triggers mtDAMP release; how immune intermediary layers—including NETosis, monocyte/macrophage metabolic reprogramming, endothelial activation, and complement—amplify the local signal into systemic inflammation; how these signals travel through the blood to injure liver, lungs, and brain; and therapeutic possibilities targeting mitochondria—via mitophagy modulation or nano-delivery—to restore barrier integrity and block systemic inflammation. By laying out a network-based “microbiota–mitochondria–barrier–multiorgan” framework, we hope to offer a deeper pathophysiological view of gut-driven organ failure and point toward promising research directions. This network-based framework is schematically depicted in **Figure 1**. In this figure, we distinguish among four categories of elements: (1) microbial metabolites (green), (2) local mitochondrial events including mtROS and intracellular signalling (yellow), (3) immune amplification mechanisms (orange), and (4) distal organ effects (blue). Solid vs. dashed arrows indicate the strength of current evidence. This visual logic is intended to clarify the network structure and evidence base, not to imply a strictly sequential pathway.

To improve readability and highlight the core questions that remain, we list the key unresolved research questions that motivate this review:

(Q1) Temporal order: Do mitochondrial changes in IECs precede barrier breakdown in humans, and if so, what is the time course? (Current animal data suggest a potential sequence, but human validation is lacking).

(Q2) Directionality: Is mitochondrial dysfunction a cause or a consequence of dysbiosis and barrier failure? (Emerging evidence supports bidirectionality, but the relative contribution of each direction remains unclear).

(Q3) Epigenetic mediation: Do acute changes in microbial metabolites during critical illness leave detectable epigenetic marks on human IECs that influence mitochondrial function?

(Q4) Quantitative thresholds: What concentrations of mtDAMPs are sufficient to trigger inflammation in distant organs, and how do portal and systemic levels relate? (Additionally, because mtDNA originates from multiple tissues, its tissue-specific contribution must be disentangled).

(Q5) Therapeutic targeting: Can interventions that stabilize mitochondrial function in IECs effectively interrupt the network from dysbiosis to MODS in humans? This network-based framework is schematically depicted in **Figure 1**.

## 2 Metabolite‑driven mitochondrial dysfunction as a gateway to epithelial barrier failure

The intestinal epithelium harbours dense microbial communities, and the metabolites they produce exert profound effects on host cell physiology (6). Among the various host–microbe interactions, communication between microbial metabolites and epithelial mitochondria is critical for maintaining gut homeostasis (6, 7). Mitochondria actively sense bacterial products and adapt their bioenergetic profiles, redox status, and biogenesis accordingly (7).

To provide a systematic overview, we summarize the major classes of microbiota-derived metabolites, their mitochondrial targets, and their downstream effects in **Table 1**. The interplay between microbial metabolites and IEC mitochondrial function has been extensively reviewed elsewhere (8). It is important to note that the effects are often concentration- and context-dependent: beneficial metabolites (e.g., SCFAs at physiological levels) promote mitochondrial health, whereas depletion of these beneficial metabolites or accumulation of harmful metabolites (e.g., excess H_2_S, aberrant secondary bile acids) drives dysfunction. This distinction between loss of protective signals and gain of damaging signals is critical for understanding the net outcome.

**Table 1:** Major microbiota-derived metabolites and their effects on intestinal epithelial mitochondria.

Metabolite class	Examples	Mitochondrial target/mechanism	Effect on mitochondrial function	Effect on barrier/inflammation	References
Short-Chain Fatty Acids (SCFAs)	Acetate, Propionate, Butyrate	TCA cycle (as fuel), HDAC inhibition	Promotes OXPHOS, ATP production; modulates membrane potential	Generally protective; enhances barrier function	(9–12)
Hydrogen Sulfide (H_2_S)	H_2_S	Cytochrome c oxidase (Complex IV), SQR	Dual: at nanomolar concentrations: antioxidant, electron donor; at high micromolar: inhibits ETC, ↑ mtROS	Dual: protective at low conc.; pro‑inflammatory at high conc.	(8, 13, 14)
Secondary Bile Acids	Deoxycholic acid (DCA), Lithocholic acid (LCA)	mPTP, apoptotic pathways	Dual: can activate apoptosis or protect from oxidative stress depending on structure/conc	Complex; can be protective or detrimental	(15–17)
Tryptophan Metabolites	Indole, Indole-3-acetic acid (IAA)	Aryl hydrocarbon receptor (AhR)	Modulates barrier function via immune signalling; effects on mitochondria not fully elucidated	Generally protective; enhances barrier	(6, 7)
Polyamines	Putrescine, Spermidine, Spermine	TCA cycle, calcium regulation	Promotes mitochondrial function and cell survival	Protective; supports barrier integrity	(15)
Trimethylamine N-oxide (TMAO)	TMAO	Complex I, III inhibition (in some cells)	Pro-inflammatory, ↑ mitochondrial dysfunction	Detrimental; may impair barrier	(16)

Abbreviations are defined in the main text or the Abbreviation list at the end of the manuscript.

SCFAs—particularly acetate, propionate, and butyrate—are abundant in the colonic lumen (9, 16). Butyrate serves as the preferred energy fuel for colonocytes: after being converted to acetyl‑CoA, it enters the tricarboxylic acid (TCA) cycle, thereby supporting oxidative phosphorylation (OXPHOS) and ATP production (3, 9). Beyond fuelling respiration, SCFAs modulate mitochondrial membrane potential and mtROS generation in a concentration‑dependent manner (7, 9). Tryptophan metabolites (e.g., indole, indole‑3‑acetic acid) influence barrier function through immune pathways (e.g., AhR signalling), but their direct effects on IEC mitochondria remain poorly characterized. Polyamines support mitochondrial function via TCA cycle and calcium regulation, while TMAO inhibits Complex I/III in some cell types, promoting dysfunction (15, 16). The effects summarized here are integrated with the evidence presented in **Table 1**.

Importantly, the communication between gut microbes and mitochondria is bidirectional (9, 18). When mitochondrial function falters, the luminal metabolic environment changes because of altered epithelial oxygen consumption and secretory patterns, favouring certain microbial communities and setting up a vicious cycle (8, 18, 19). This feedback loop is especially relevant in critical illness, where dysbiosis and mitochondrial damage often coexist (19, 20). Thus, it is more accurate to view the microbiota–mitochondria relationship as a dynamic network rather than a unidirectional driver. In summary, microbial metabolites directly govern the functional state of intestinal epithelial mitochondria (8, 9). As the microbial community shifts towards a pro‑inflammatory, metabolite‑depleted profile (loss of SCFAs and gain of excess H_2_S or aberrant bile acids), mitochondrial function declines—preparing the ground for the disruptive events described next.

## 3 Mitochondrial dysfunction disrupts the intestinal barrier via energy failure, mtROS, calcium dysregulation, and mtDAMP‑driven inflammation

Once microbial metabolites compromise mitochondrial function, the intestinal epithelium faces a series of destructive events. The earliest and most direct consequence is energy failure. Tight junctions—the core structures of the epithelial barrier—depend heavily on ATP for their assembly, maintenance, and repair. When mitochondrial OXPHOS becomes inefficient and ATP production drops, the stability and localization of key tight‑junction proteins such as zonula occludens-1 (ZO-1) and occludin are affected—existing studies more commonly report functional changes or altered membrane localization rather than purely transcriptional downregulation (8, 18). As a result, intercellular contacts loosen, paracellular permeability increases, and luminal contents leak into the interstitial space.

A second, equally important pathway involves mtROS. Under normal conditions, low‑level mtROS production during electron transfer acts as an intracellular signalling mechanism that helps preserve barrier function. However, when mitochondria are damaged or metabolic substrates are abnormal, mtROS production rises sharply, overwhelming cellular antioxidant defences. Excessive mtROS activate the nuclear factor‑κB (NF‑κB) and mitogen-activated protein kinase (MAPK) pathways, which then directly phosphorylate tyrosine or serine/threonine residues on tight‑junction proteins, triggering their internalisation from the plasma membrane and subsequent degradation (8, 18). The loss of ZO‑1 and occludin leaves tight‑junction strands fragmented and discontinuous.

A third damaging mechanism involves calcium dysregulation. Mitochondria buffer and regulate intracellular calcium. Under pathological conditions, collapse of the mitochondrial membrane potential and aberrant opening of the mitochondrial permeability transition pore (mPTP) impair calcium uptake, leading to elevated cytosolic calcium. This activates calpain—a calcium‑dependent cysteine protease (19, 21)—which directly cleaves E‑cadherin (21). E‑cadherin is essential for epithelial polarity and adherens junctions; its degradation disrupts the structural integrity of the epithelium, moving barrier failure from “loosened connections” to “architectural disintegration” (19). Experimental evidence supporting the role of calpain in barrier regulation comes from studies showing that occludin proteolysis is sensitive to calpain inhibition, and that increased calpain activity correlates with tight junction disruption in intestinal epithelial cells (22).

When mitochondrial damage reaches a certain threshold, damaged mitochondria release danger signals—mtDNA and mtROS—directly into the cytoplasm. Cytosolic mtDNA is recognised by cGAS, activating the STING pathway and inducing type I interferons and pro‑inflammatory cytokines (15, 23). Simultaneously, mtROS and mtDNA activate the NLRP3 inflammasome, leading to secretion of interleukin‑1β (IL‑1β) and interleukin‑18 (IL‑18) (20, 24). It is crucial to distinguish these two layers of mtDAMP action: (1) the intracellular sensing in IECs, where mtDNA activates cGAS‑STING and NLRP3 to drive local inflammation and barrier disruption; and (2) the systemic action of circulating mtDNA, which acts as a DAMP on organ‑resident immune cells. These two layers are distinct but interconnected, as local inflammation can amplify the release of mtDAMPs into the circulation. The relative contributions of the cGAS‑STING versus NLRP3 pathways in stressed intestinal epithelial cells remain to be determined. These inflammatory cytokines act on neighbouring intestinal epithelial cells and immune cells, amplifying local inflammation and feeding back to further suppress mitochondrial function—creating a positive feedback loop (20).

Beyond the direct effects on tight junctions, mitochondrial dysfunction may also compromise barrier integrity by affecting secretory epithelial cell lineages. Paneth cells and goblet cells, which produce antimicrobial peptides and mucus, respectively, are highly dependent on OXPHOS for their function (8). Disruption of mitochondrial function in these cells could lead to a depleted mucus layer and reduced antimicrobial defence, further increasing the risk of bacterial translocation and systemic inflammation. This additional mechanism broadens the scope of mitochondrial contribution to barrier failure beyond the paracellular route.

The local inflammatory response is further amplified by immune intermediary mechanisms. NET formation can be triggered by mtDAMPs and microbial products; NETs, decorated with histones and proteases, further damage endothelial cells and promote microvascular thrombosis, exacerbating tissue ischemia and facilitating systemic dissemination of inflammatory signals (25, 26). Concurrently, monocytes and macrophages undergo metabolic reprogramming—shifting from oxidative phosphorylation to glycolysis—which sustains a pro‑inflammatory phenotype and amplifies cytokine and ROS production, creating a self‑perpetuating inflammatory loop (27). Endothelial activation, characterized by upregulation of adhesion molecules (e.g., intercellular adhesion molecule 1 [ICAM-1], vascular cell adhesion molecule 1 [VCAM-1]) and increased vascular permeability, allows inflammatory cells and mediators to extravasate into parenchymal organs, bridging local gut inflammation to systemic organ injury (28). Complement activation (e.g., C3a, C5a) may further amplify this response, although direct evidence linking gut-derived mtDAMPs to complement in MODS is currently sparse.

In summary, energy depletion, oxidative stress, calpain activation, and mtDAMP release do not operate in isolation; they intertwine and reinforce each other. The intestinal barrier thus transforms from a passive “leaky” structure into an active amplifier that generates and releases inflammatory signals—the critical turning point from a local defect to a systemic inflammatory storm (8, 18, 20).

## 4 From the gut–liver axis to causal network validation: gaps, trackers, and testable predictions

### 4.1 The gut–liver axis as a natural testbed for the network hypothesis

The portal vein carries blood directly from the intestinal circulation to the liver, making the liver the first solid organ to be exposed to mtDAMPs and microbial metabolites that have leaked through a compromised barrier. Thus, the gut–liver axis offers a natural experimental setting for testing whether the “microbiota–mitochondria–barrier” triad genuinely drives distant organ injury.

Once mtDNA enters the portal circulation and reaches the liver sinusoids, Kupffer cells—the liver’s resident macrophages—which express a wide range of pattern‑recognition receptors, encounter these signals. mtDNA binds to TLR9 on Kupffer cells, which induces the production and release of pro‑inflammatory cytokines including tumour necrosis factor‑α (TNF‑α), interleukin‑6 (IL‑6), and IL‑1β (29). Concurrently, locally generated mtROS activate the NLRP3 inflammasome in hepatic stellate cells and hepatocytes, promoting sterile inflammation that can progress from simple steatosis to steatohepatitis and, in severe cases, to acute‑on‑chronic liver failure (30, 31). Supporting this causal interpretation, clinical and experimental evidence shows that mtDNA release from injured hepatocytes drives liver inflammation via TLR9 activation, and plasma mtDNA levels correlate positively with elevated transaminases and markers of liver dysfunction (32, 33).

Notably, the gut–liver axis also involves a retrograde feedback loop. The injured liver releases mediators such as bile acids, cytokines, and mtROS (which act locally), which can return to the gut via the biliary tract and systemic circulation. These mediators further impair mitochondrial function in intestinal epithelial cells, thereby closing a loop that locks the gut and liver into a self‑amplifying cycle (34). The existence of this two‑way communication strengthens the idea that mitochondrial dysfunction is not merely an epiphenomenon but a genuine mechanistic hub within a broader network of direct and reciprocal interactions.

### 4.2 The causal network under scrutiny: gaps and the promise of circulating trackers

Although each segment of the “microbiota–mitochondria–barrier–multiorgan” network is supported by evidence, moving from correlation to causation requires addressing several critical gaps that distinguish direct causality from mere association or feedback regulation. At the same time, the network yields measurable predictions that can be tested using circulating and tissue‑based markers. This section examines the major unresolved questions and outlines how candidate trackers could convert the causal framework into a testable, quantitative model.

#### 4.2.1 What remains to be proven

Each piece of the proposed network has biological plausibility, but turning a correlative model into a validated mechanistic network demands that we confront several gaps in human evidence.

##### 4.2.1.1 Temporal order (Q1)

Most human studies measure microbiota, mitochondrial function, and barrier permeability at a single time point. That makes it impossible to know whether mitochondrial dysfunction comes before barrier breakdown or the other way around (8, 18). Animal data from different models have reported changes at various time points, but these data come from separate studies using different experimental conditions, and no single study has established a unified temporal sequence from metabolite change to barrier disruption. Therefore, we do not propose a specific 6–36 h timeline for humans. Directly extrapolating these disparate mouse-derived findings to human critical illness is problematic due to substantial differences in physiology, disease progression, and temporal dynamics. What we need are prospective cohort studies with serial sampling—every 6 to 12 hours after intensive care unit (ICU) admission—to see if mitochondrial changes consistently precede measurable increases in gut permeability in humans (3, 5).

##### 4.2.1.2 Directional causality (Q2)

Forward experiments (faecal transplantation or metabolite supplementation) can partially restore mitochondrial function and barrier integrity (8, 35). That much is clear. Reverse experiments—where we induce a defined mitochondrial defect in healthy animals and then watch what happens to the microbiota—are far rarer. A few groups have done this: Urbauer et al. showed that intestinal epithelial‑specific mitochondrial perturbation causes microbiota‑dependent injury (18); Alula et al. found that the interplay between gut microbes and host epithelial mitochondrial dysfunction is necessary for spontaneous intestinal inflammation (19). Still, such reverse‑direction studies are too few to fully establish bidirectionality. More are needed. Moreover, the relative contribution of each direction in human pathophysiology remains unknown.

##### 4.2.1.3 Epigenetic mediation (Q3) —largely unexplored in humans

There is evidence from cell lines and short‑term animal studies that microbial metabolites like δ‑valerobetaine, H_2_S, or secondary bile acids can regulate mitochondrial genes through DNA methylation or histone modifications (16). But whether acute changes in these metabolites during critical illness leave detectable epigenetic marks on human IECs—and whether those marks correlate with long‑term outcomes—remains a black box (16, 34). One feasible approach would be to capture shed intestinal epithelial cells from faecal samples or endoscopic brushing, then correlate their methylation status with prior plasma metabolite levels. Until someone does those experiments, epigenetics as a mechanistic link in our network is speculative (16).

##### 4.2.1.4 Quantitative thresholds (Q4)

We know that plasma mtDNA levels correlate with MODS severity (29, 32). But we lack precision on several quantitative relationships: (a) what concentration of mtDAMPs in portal blood is enough to trigger liver inflammation? (32, 33); (b) how do mtDAMP levels in portal versus systemic circulation relate over time? (29, 32); (c) what increase in intestinal permeability (e.g., lactulose/mannitol ratio) translates into clinically meaningful distant organ injury? (3, 18). Furthermore, because circulating mtDNA originates from multiple tissues (immune cells, skeletal muscle, liver, etc.), its elevation reflects systemic damage rather than gut‑specific injury; thus, its interpretation requires additional context, such as tissue‑specific markers or temporal dynamics, to be used as a gut‑derived signal. Answering these will require time‑resolved studies that simultaneously measure gut permeability, portal or systemic mtDNA, and organ dysfunction scores—ideally in patients undergoing portal vein catheterisation or abdominal surgery (29, 32, 34).

To be honest, until we fill these four gaps—temporal order, directionality, epigenetics, and thresholds—the “microbiota–mitochondria–barrier–multiorgan” network remains a well‑grounded hypothesis rather than an established human pathophysiology (3, 8, 18). We reiterate: this is a testable hypothesis, not a proven mechanism.

#### 4.2.2 Circulating metabolites and mtDNA as candidate monitoring biomarkers

The network model offers hypotheses that can be tested by dynamically tracking circulating markers. One hypothesis is that a decline in protective microbial metabolites (e.g., δ‑valerobetaine) might precede elevation of plasma mtDNA, which could in turn precede clinical signs of organ dysfunction. However, this temporal sequence has not been validated in humans. Importantly, circulating mtDNA alone cannot validate the proposed gut-derived mechanism because it originates from multiple tissues (e.g., immune cells, skeletal muscle, liver) (29, 32). To improve tissue-origin attribution, future studies should consider: (1) tissue-specific methylation signatures of circulating mtDNA, (2) extracellular vesicle profiling to identify gut-derived vesicles, or (3) combined use of organ-specific injury markers (e.g., transaminases for liver, creatinine for kidney). These approaches remain exploratory and require methodological validation. To support this logic, a recent study tracked the relationship between a specific microbial metabolite, δ‑valerobetaine (δ‑VB), and intestinal barrier integrity. Askew and colleagues found that δ‑VB strengthens the intestinal epithelial barrier by decreasing permeability and promoting wound healing (35). Although not yet performed in a longitudinal sepsis cohort, their work conceptually validates the use of circulating metabolite levels as dynamic readouts of barrier status, setting the stage for future time‑resolved studies that could track the sequence from metabolite decline to mtDNA elevation to MODS.

To address Q1 (temporal order), prospective cohort studies with dense serial sampling (e.g., every 6–12 hours) are essential. For Q2 (directionality), animal models with inducible, cell‑type‑specific mitochondrial defects—such as the intestinal epithelial‑specific mitochondrial perturbation used by Urbauer et al. (18)—should be coupled with longitudinal tracking of faecal metabolites and circulating mtDNA to capture the reverse causal direction. For Q3 (epigenetic mediation), shed intestinal epithelial cells can be captured from faecal samples or by endoscopic brushing; their methylation status at promoters of mitochondrial biogenesis genes can be correlated with antecedent plasma levels of candidate metabolites (16). For Q4 (quantitative thresholds), patients undergoing abdominal surgery or transhepatic portal vein catheterisation would offer a valuable opportunity to measure portal versus systemic mtDNA gradients. Such measurements could help define the threshold amounts of mtDAMP leakage required to trigger distal organ injury, though direct clinical evidence specifically linking portal venous mtDNA levels to postoperative liver dysfunction is still lacking.

Together, these circulating and tissue‑based trackers offer a practical route to convert the causal network from a conceptual framework into a testable, quantifiable model. Filling the four gaps will require well‑designed longitudinal human studies and targeted animal experiments, but the tools and candidate markers are already at hand.

### 4.3 A common mechanistic hub across the gut–liver, gut–lung, and gut–brain axes

Although the liver, lung, and brain differ in their cellular composition and vulnerability, the pathways by which a leaky gut injures these distant organs converge on a shared set of molecular events. Recent comprehensive reviews have systematically mapped organ crosstalk in sepsis, covering the gut‑microbiome‑liver‑brain axis among others (36). However, these broader frameworks have not specifically focused on the mitochondrial/mtDAMP‑centred mechanism proposed here. In each case, the triad of mtDAMPs (primarily mtDNA and locally generated mtROS), microbial metabolites that have entered the circulation, and the host’s innate immune sensors forms the core of the response (8, 16).

The local inflammatory response in the gut, triggered by mtDAMP release, activates several intermediary immune mechanisms. NET formation, driven by mtDAMPs and microbial products, damages endothelial cells and promotes microvascular thrombosis (25, 26). Monocytes and macrophages undergo metabolic reprogramming toward glycolysis, sustaining a pro-inflammatory phenotype and amplifying cytokine and ROS production (27). Endothelial activation, with upregulated adhesion molecules (ICAM-1, VCAM-1), increases vascular permeability (28). Complement activation (e.g., C3a, C5a) may further amplify this response, although direct evidence linking gut-derived mtDAMPs to complement in MODS is currently sparse. These mechanisms, supported primarily by cell and animal studies, are not mere downstream consequences but active amplifiers that facilitate systemic dissemination of inflammatory signals.

When mtDNA reaches the circulation, it engages TLR9 on tissue-resident macrophages in distal organs; however, the consequences are shaped by organ-specific immune environments. In the liver, Kupffer cell TLR9 activation induces TNF-α, IL-6, and IL-1β (29), while locally generated mtROS activate NLRP3 in hepatic stellate cells (30, 31); Kupffer cell-derived cytokines and NETs can exacerbate hepatocellular damage. In the lung, alveolar macrophage TLR9 and NLRP3 activation contribute to acute respiratory distress, with endothelial activation increasing alveolar-capillary permeability. In the brain, microglial TLR9 activation and compromised blood–brain barrier facilitate neuroinflammation, potentially contributing to sepsis-associated encephalopathy. The shared core mechanism—mtDAMP → TLR9/NLRP3 → sterile inflammation—is thus modulated by organ-specific immune milieus and amplification pathways. This integrated view supports the development of systemic mitoprotective strategies rather than organ-specific approaches.

Third, microbial metabolites that escape through the broken barrier create an organ‑specific but mechanistically similar milieu. For example, high levels of H_2_S or abnormal secondary bile acids in portal blood directly impair hepatocyte mitochondria; in the systemic circulation, they can reach the pulmonary endothelium and the brain, where they similarly inhibit complex IV and trigger mtROS bursts (8, 13, 16). The difference among organs is largely one of exposure dose and duration, not of fundamental mechanism. However, this conclusion—that the axes are primarily quantitatively rather than qualitatively different—is a hypothesis that requires further validation. Given the substantial differences in cellular composition, tissue architecture, and immune environments across these organs, a more cautious interpretation is warranted. It is plausible that while the core molecular triad (mtDAMP → TLR9/NLRP3 → sterile inflammation) is shared, organ‑specific responses and thresholds may lead to qualitatively distinct injury patterns. For instance, the brain’s unique sensitivity to oxidative stress and the liver’s high metabolic activity could render them differentially susceptible to mtDAMP‑mediated damage.

Differences across axes are likely quantitative rather than strictly qualitative, though this remains to be confirmed. In the gut–lung axis, a disrupted intestinal barrier allows mtDAMPs and microbial metabolites to enter the systemic circulation and reach the pulmonary vasculature, where they trigger alveolar inflammation and worsen acute lung injury. Experimental evidence from severe acute pancreatitis indicates that intestinal barrier damage contributes directly to systemic inflammatory response syndrome (SIRS) and acute lung injury, supporting a gut‑driven pathway to respiratory failure (5). In the gut–brain axis, mitochondrial signals may bypass the blood–brain barrier or act via the vagus nerve, potentially contributing to delirium and sepsis‑associated encephalopathy (15). However, direct evidence for mtDAMP‑mediated brain injury via the vagus nerve in humans is still limited. Nevertheless, the underlying triad—mtDAMP release, TLR9/NLRP3 activation, and sterile inflammation—operates in all three territories.

Recognising this common triad—mtDAMP → TLR9/NLRP3 → sterile inflammation—unifies the gut–liver, gut–lung, and gut–brain axes under a single therapeutic rationale: interventions that reduce mtDNA release, scavenge mtROS, or block NLRP3 should protect all three organs simultaneously (20). Furthermore, targeting immune intermediary steps (e.g., NETosis inhibitors, metabolic modulators of macrophages, endothelial stabilizers, complement inhibitors) could provide additional leverage. This cross‑axis convergence supports the development of systemically acting mitoprotective strategies, rather than organ‑specific approaches, for patients at risk of multiorgan dysfunction after intestinal barrier failure.

## 5 Discussion and translational perspectives

### 5.1 Therapeutic strategies and evidence maturity

**Figure 2:**
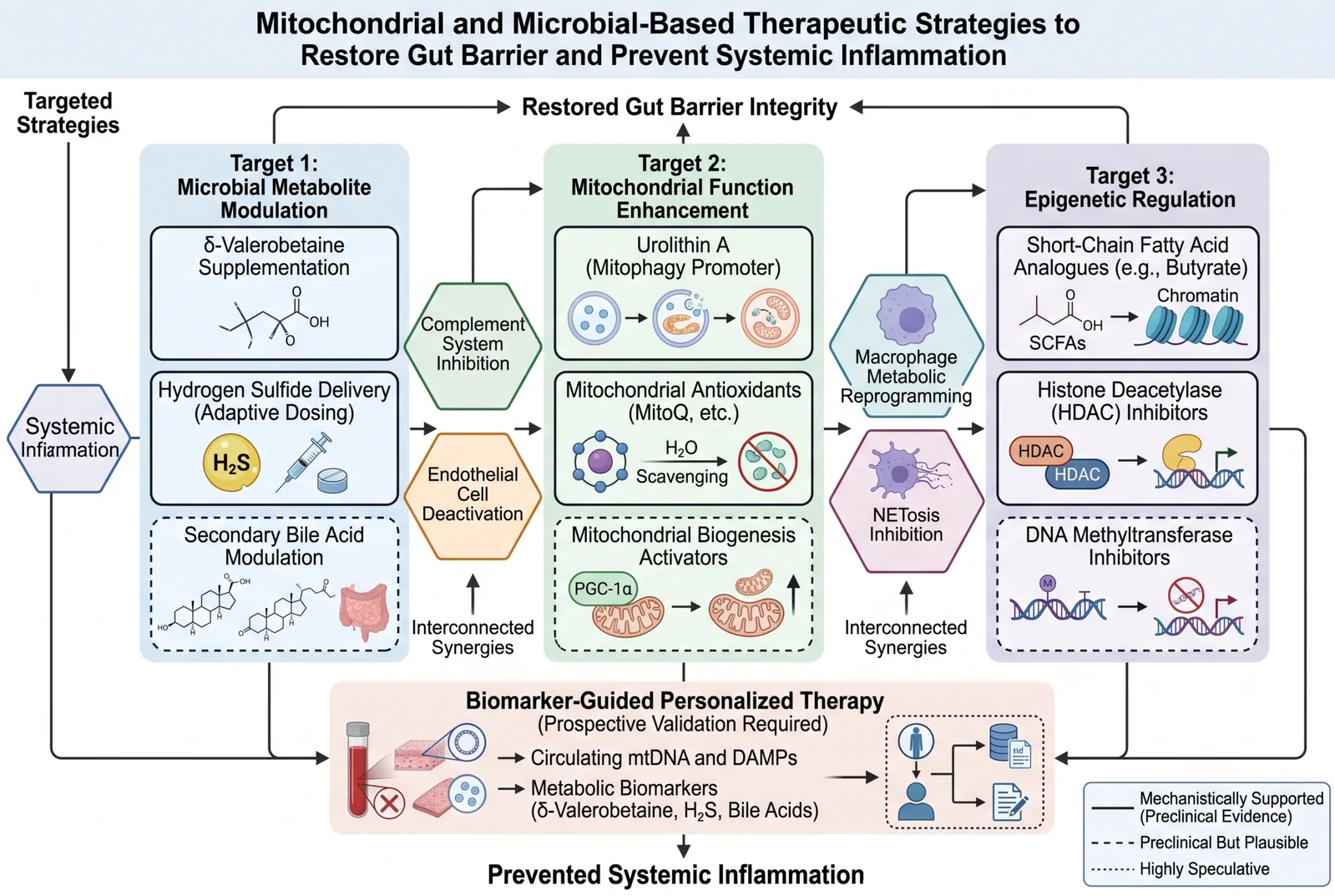
Multi‑dimensional intervention strategies targeting the gut–mitochondria–barrier network. Therapeutic strategies can target three interconnected nodes: microbial metabolites (δ-valerobetaine supplementation, hormetic H_2_S delivery), mitochondrial function (mitophagy enhancers like urolithin A, mitochondrial antioxidants), and epigenetic regulation (SCFA analogues, HDAC inhibitors). Immunomodulatory approaches targeting NETosis, macrophage metabolism, endothelial activation, or complement could complement mitoprotective strategies. Biomarker‑guided therapy—based on circulating mtDNA and metabolite levels (δ‑valerobetaine, H_2_S, secondary bile acids)—is a research concept that requires prospective validation. Interventions are categorized by evidence maturity: “mechanistically supported” (solid outlines, preclinical evidence), “preclinical but plausible” (dashed outlines), and “highly speculative” (dotted outlines). This framework aims to restore intestinal barrier integrity and prevent systemic inflammation.

If the three dimensions—microbial metabolites, mitochondrial function, and epigenetic regulation—indeed form an integrated network, then optimal therapy should target more than one node at a time. However, we must be clear about the maturity of evidence for each proposed intervention. To provide clarity, we have stratified therapeutic strategies into three categories based on the strength of supporting evidence. The multi‑dimensional intervention strategies are summarized in **Figure 2**.

#### 5.1.1 Mechanistically supported (preclinical/animal evidence)

SCFA analogues (e.g., tributyrin)—preclinical studies show they upregulate mitochondrial biogenesis genes and preserve tight‑junction proteins (10, 37). Mitochondria‑targeted antioxidants (e.g., MitoQ)—MitoQ has been shown to modulate lipopolysaccharide‑induced intestinal barrier dysfunction via activating the nuclear factor erythroid 2-related factor 2 (Nrf2) signalling pathway (38), and to protect the intestinal barrier by ameliorating mitochondrial DNA damage via the Nrf2/antioxidant response element (ARE) signalling pathway (39). Mitophagy enhancers (e.g., urolithin A)—urolithin A improves autophagy flux and prevents mitochondrial and muscle impairments in sepsis models (40), and alleviates sepsis‑induced acute lung injury by reducing mitochondrial dysfunction and modulating macrophage polarisation (41).

#### 5.1.2 Preclinical but plausible

Controlled H_2_S donors (e.g., GYY4137)—GYY4137 has been shown to donate electrons to the mitochondrial electron transport chain (ETC) via sulfide:quinone oxidoreductase (SQR) (14) and to ameliorate mitochondrial damage and mtDNA injury in experimental models (42). This “hormetic H_2_S” approach is promising but requires further validation in models of intestinal barrier injury. Combination SCFA analogues and histone deacetylase (HDAC) inhibitors—synthetic SCFA analogues combined with low‑dose HDAC inhibitors such as valproate or entinostat show synergistic effects in colitis models (10, 37), but this has not been tested in the context of MODS or systemic injury.

#### 5.1.3 Highly speculative

Epigenetic–mitochondrial nanodrugs—nanoparticles co‑delivering a DNA methyltransferase inhibitor and a mitochondria‑targeted antioxidant to IECs represent a technologically advanced but largely untested concept with significant delivery and safety hurdles. Multi‑biomarker‑guided precision intervention—while conceptually appealing, this strategy requires prospective validation of biomarker panels and the establishment of clinically meaningful thresholds, which are currently lacking.

In addition to mitochondria-targeted interventions, immunomodulatory approaches targeting these intermediary amplifiers—such as NETosis inhibitors (e.g., peptidylarginine deiminase 4 [PAD4] inhibitors), macrophage metabolic modulators (e.g., 2-deoxy-D-glucose [2-DG] or metformin), endothelial stabilizers (e.g., angiopoietin-1 mimetics), or complement inhibitors—have shown promise in preclinical sepsis models (26–28). However, these strategies remain experimental and have not been tested specifically in the context of gut‑derived MODS.

The therapeutic discussion must also consider broader immunological effects, as microbial metabolites influence numerous pathways beyond mitochondrial regulation, including interleukin‑22 (IL‑22)‑mediated protective responses. Any strategy targeting mitochondria should be evaluated in the context of these pleiotropic effects.

### 5.2 Mitohormesis as a unifying hypothesis

The concept of mitohormesis serves as a testable unifying hypothesis for integrating these interventions, although direct clinical evidence in the context of intestinal barrier protection remains lacking. The beneficial effects of low‑grade mitochondrial stress have been systematically reviewed, with evidence showing that repeated exposure to mild mitochondrial stress induces adaptive responses that build resilience against higher levels of stress (43). The epigenetic dimension adds a layer of plasticity: short‑term metabolic stress can produce long‑lasting changes in gene expression, raising the possibility that a well‑timed, mild mitochondrial stress—a hormetic stimulus—might trigger epigenetic adaptations that prepare the intestinal barrier to withstand more severe injury later on. It has been reported that caloric restriction, intermittent fasting, and certain plant polyphenols (such as resveratrol and sulforaphane) activate the Nrf2‑ARE pathway. However, direct evidence linking these compounds to mitophagy specifically in the context of intestinal barrier protection is still lacking, and the connection to the proposed microbiota–mitochondria–barrier axis remains insufficiently developed. Further mechanistic studies are needed to clarify how mitohormetic responses support, modify, or challenge the central causal model.

### 5.3 Clinical translation roadmap

It must be emphasised that the following steps are research proposals, not clinical algorithms. Each requires prospective validation before any clinical application. To move the causal network model from a conceptual framework toward clinical application, three concrete steps deserve near‑term attention. First, biomarker validation—existing prospective sepsis/MODS cohorts with stored plasma samples can be used to retrospectively test the predicted temporal sequence (protective metabolite decline → mtDNA elevation → organ dysfunction). Measuring δ‑valerobetaine, H_2_S, secondary bile acids, and circulating mtDNA in serial samples would establish whether the animal‑derived time course holds in humans. Crucially, for mtDNA measurements, authors should specify the target (e.g., total cell‑free mtDNA, copy‑number normalized mtDNA, or specific quantitative polymerase chain reaction [qPCR] targets such as NADH dehydrogenase subunit 1 [ND1] or cytochrome B) and discuss potential confounders such as haemolysis, platelet activation, leukocyte lysis during processing, renal/hepatic clearance, and timing of sampling. Second, exploratory risk stratification—a composite score combining low δ‑valerobetaine and high mtDNA could be derived and tested retrospectively in existing cohorts for its association with subsequent MODS development. However, this is a research tool, not a clinical algorithm. Any predictive performance would require prospective validation, and the score’s utility is contingent on resolving the tissue-origin ambiguity of mtDNA. As such, it should not be interpreted as enabling early clinical triage or preventive intervention at this stage. For clinical anchoring, this biomarker strategy should be tied to serial Sequential Organ Failure Assessment (SOFA) trajectories, as SOFA‑based risk stratification has clinical relevance in severe acute pancreatitis and MODS (44). Third, proof‑of‑concept trials—once a high‑risk signature is validated, a small randomised controlled trial could compare a hormetic enteral formula (e.g., enriched with resveratrol or sulforaphane) against standard feeding in patients with the signature. The primary outcomes would be mechanistic (change in plasma mtDNA, intestinal permeability markers) rather than clinical, providing a rapid test of the mitohormesis rationale before larger outcome trials. These steps are feasible with existing technologies and collaborative critical care networks, and they would directly address Gap 4 (quantitative thresholds) identified in section 4.2.1.

To further aid readers in distinguishing established evidence from the speculative elements of the hypothesis, we provide a structured evidence summary in **Table 2**.

**Table 2:** Evidence supporting each step of the proposed “microbiota–mitochondria–barrier–multiorgan” causal cascade.

Step in causal chain	Evidence level	Key supporting studies (examples)	Key gaps/uncertainties
1. Dysbiosis → Altered Metabolites	Strong (Human & Animal)	(6, 7, 9)	Specific metabolite changes in early MODS not fully characterized.
2. Altered Metabolites → IEC Mitochondrial Dysfunction	Moderate (Cell & Animal)	(8, 13, 14, 18)	Direct causal evidence in human IECs is limited. Concentration-dependent effects (e.g., H_2_S) are complex.
3. IEC Mitochondrial Dysfunction → Barrier Breakdown	Moderate (Cell & Animal)	(8, 18, 19, 22)	Relative contribution of ATP depletion, ROS, and calpain in humans is unclear. Role of secretory cells needs further study. Transcriptional vs. functional changes in tight junction proteins require clarification.
4. Barrier Breakdown → mtDAMP Release (Local)	Moderate (Cell & Animal)	(15, 20, 23, 24)	Relative roles of cGAS-STING vs. NLRP3 in IECs are not fully defined.
5. Local Inflammation → Immune Amplification (NETs, Macrophage Reprogramming, Endothelial Activation)	Emerging (Cell & Animal)	(25–28)	Direct evidence linking gut‑derived mtDAMPs to these intermediary mechanisms in human MODS is sparse. Quantitative contribution of each amplifier is unknown.
6. mtDAMP Release → Systemic Inflammation & Organ Injury	Moderate (Human & Animal)	(29, 32, 33)	Temporal order, dose‑response, portal vs. systemic gradients, and tissue‑origin specificity need validation in humans.
7. Epigenetic Mediation	Weak/Speculative (Cell)	(16, 34)	Almost entirely unexplored in human MODS.
8. Therapeutic Targeting (Mitochondria)	Preclinical (Animal/Cell)	(10, 14, 37–41)	No clinical trials have tested mitochondrial‑targeted therapies to prevent MODS via barrier protection.

## 6 Conclusions and future perspectives

We have proposed a dynamic network in which gut microbiota alterations, mitochondrial dysfunction, barrier breakdown, immune amplification, and systemic inflammation are interconnected. This framework emphasizes that mitochondrial dysfunction in IECs may serve as a mechanistic hub linking microbial imbalance to remote organ damage within a dynamic, bidirectional system. For the clinician, three points stand out.

First, a negative blood culture does not rule out gut‑driven inflammation. Even without overt bacterial translocation, damaged IECs release mtDNA and locally generated mtROS into the circulation. These endogenous danger signals can trigger and sustain systemic inflammation. Measuring circulating mtDNA—which correlates with liver injury and MODS severity—may offer a more direct indicator of gut‑derived inflammation than traditional endotoxin assays, but clinicians must recognize that mtDNA is not gut‑specific; its elevation reflects systemic cellular damage, and its interpretation requires integration with other clinical and laboratory data to infer gut origin.

Second, dynamic tracking of plasma metabolites could buy time before MODS sets in. If the proposed temporal sequence holds (a drop in protective metabolites like δ‑valerobetaine, followed by a rise in mtDNA, followed by clinical organ failure), then serial blood sampling might identify patients who are about to deteriorate 24–48 hours before conventional criteria are met. This could open a window for early, targeted interventions—for example, adjusting enteral nutrition or adding gut‑protective measures—rather than waiting for organ failure to declare itself. However, this remains a hypothesis that requires prospective validation.

Third, “mitohormetic” enteral formulas are a testable hypothesis, not yet a clinical recommendation. The idea that mild, timed mitochondrial stress (e.g., using resveratrol or sulforaphane in feeding formulas) could prepare the intestinal barrier to withstand later injury is biologically plausible but unproven in patients. At this stage, it remains a speculative hypothesis. It provides a rationale for small proof‑of‑concept trials in well‑defined high‑risk populations, but should not be interpreted as a clinical recommendation or practice change. These three takeaways do not replace the need for more human data. As discussed in section 4.2.1, we still lack definitive evidence on temporal order, directionality, human epigenetic mediation, and quantitative thresholds. Filling these gaps will require prospective cohorts and targeted trials. Nevertheless, the framework outlined here offers a way to move beyond the old picture of the gut as a passive leak and toward a more precise, mechanism‑based understanding of MODS.
